# Supplementary figures and images for: Time-trend in excess weight in Brazilian adults: A systematic review and meta-analysis
Source: PLoS One. 2021 Sep 28;16(9):e0257755. doi: 10.1371/journal.pone.0257755 (PMC8478247; doi:10.1371/journal.pone.0257755)

**S1 Fig.** Funnel plot of excess weight prevalence in Brazilian adults, from 1974 until 2020


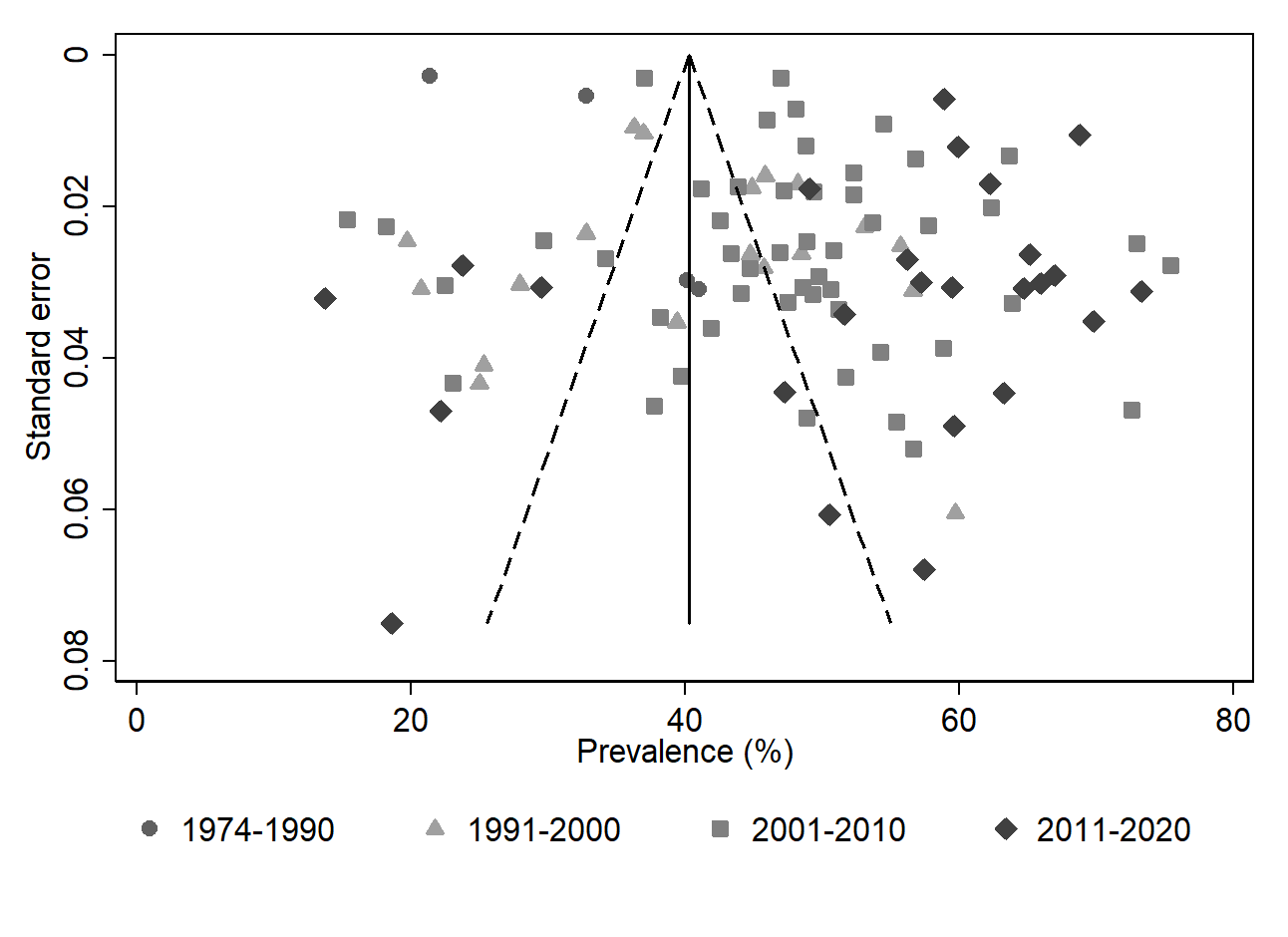

Supplement: S1 Fig — (DOCX) [file pone.0257755.s001.docx]

**S2 Fig.** Funnel plot of overweight prevalence in Brazilian adults, from 1974 until 2020.


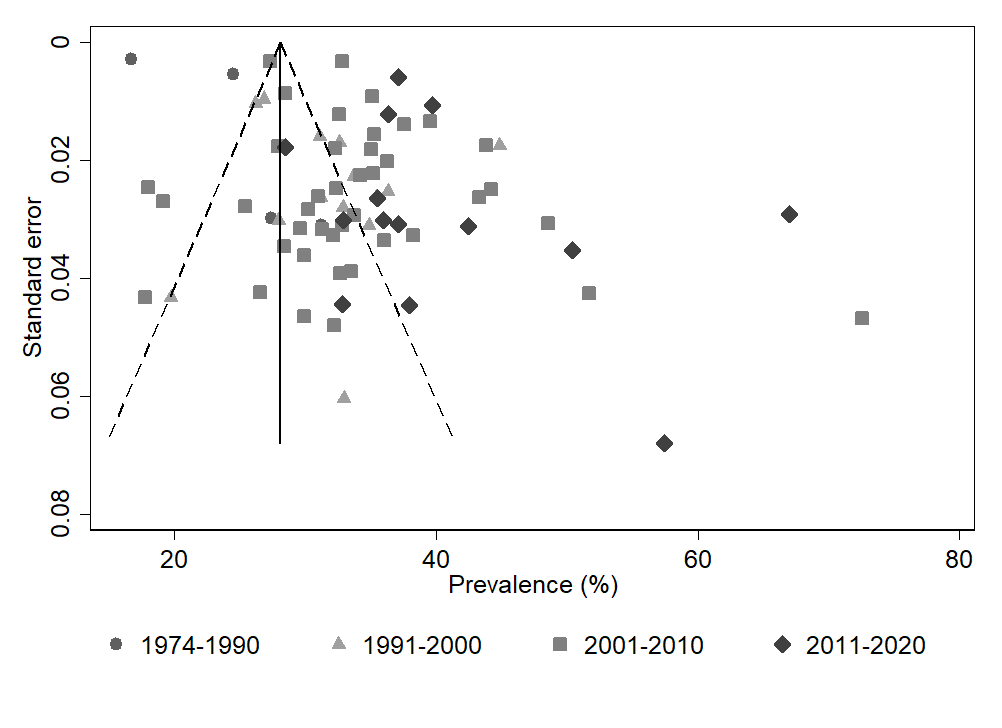

Supplement: S2 Fig — (DOCX) [file pone.0257755.s002.docx]
