# Supplementary material for: Time-trend in excess weight in Brazilian adults: A systematic review and meta-analysis
Source: PLoS One. 2021 Sep 28;16(9):e0257755. doi: 10.1371/journal.pone.0257755 (PMC8478247; doi:10.1371/journal.pone.0257755)
Supplement: S3 Fig — (DOCX) [file pone.0257755.s003.docx]

**S3 Fig.** Funnel plot of obesity prevalence in Brazilian adults, from 1974 until 2020.


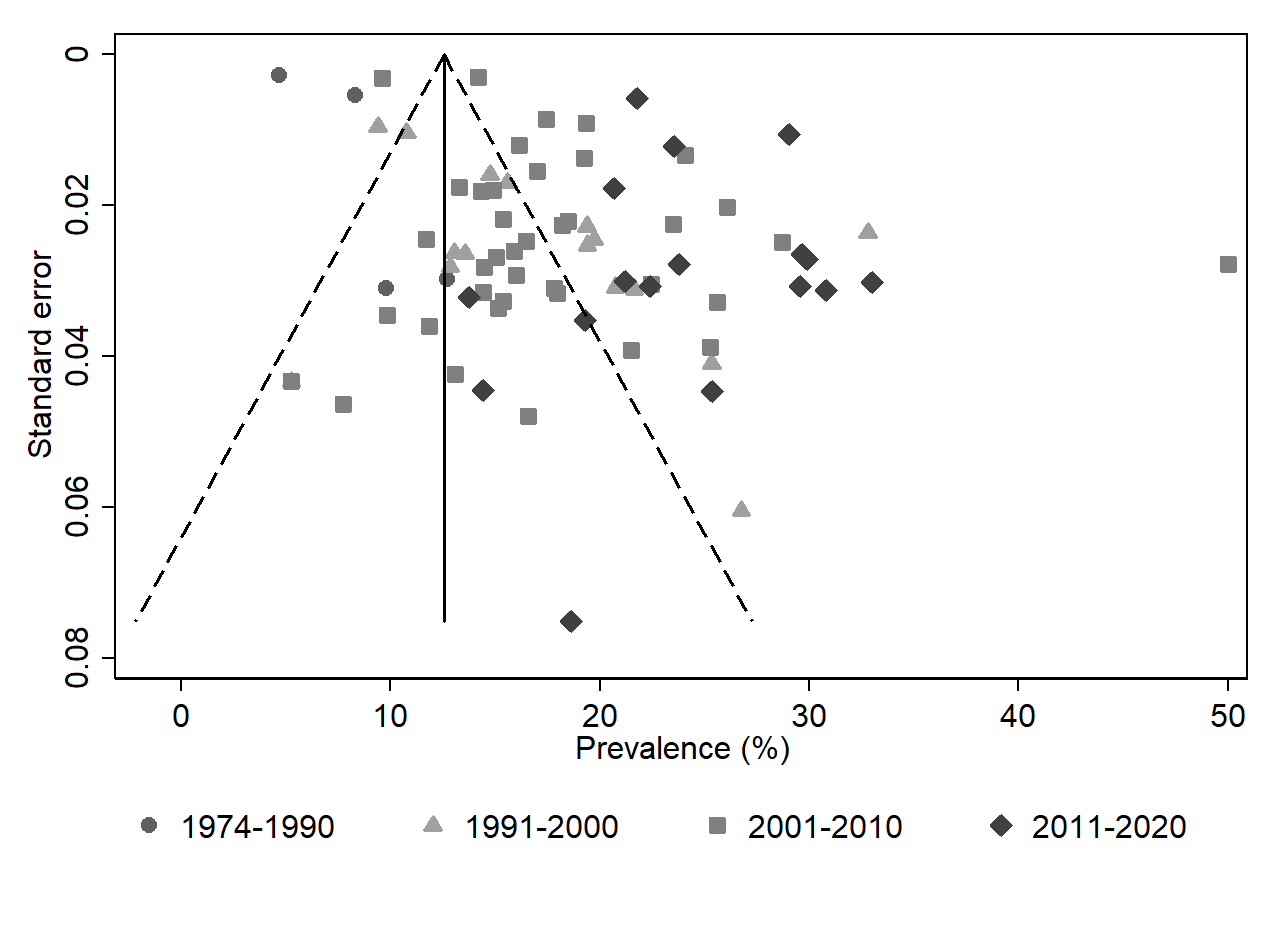


References

1. Lino MZR, Muniz PT, Siqueira KS. Prevalência e fatores associados ao excesso de peso em adultos: inquérito populacional em Rio Branco, Acre, Brasil, 2007-2008. Cad Saúde Pública. 2011;27:797-810.

2. Cordeiro J, Freitas S. Prevalência e fatores associados ao excesso de peso em uma população urbana do interior do Amazonas, Brasil. Rev Bras Prom Saúde. 2016;29:533-43.

3. Costa EC, Lira PIC, Oliveira JS, Menezes RCE, Tavares FCLP, Batista Filho M. Evolução do excesso de peso e fatores associados em mulheres de 10 a 49 anos em Pernambuco, Nordeste, Brasil. Rev Nutr. 2014;27:513-24.

4. Florencio TM, Ferreira HS, de Franca AP, Cavalcante JC, Sawaya AL. Obesity and undernutrition in a very-low-income population in the city of Maceio, northeastern Brazil. Br J Nutr. 2001;86(2):277-84.

5. Lessa I, Magalhães L, Araújo MJ, Almeida Filho N, Aquino E, Oliveira MMC. Hipertensão arterial na população adulta de Salvador (BA) - Brasil. Arq Bras Cardiol. 2006;87:747-56.

6. Oliveira LPM, Assis AMO, Silva MCM, Santana MLP, Santos NS, Pinheiro SMC, et al. Fatores associados a excesso de peso e concentração de gordura abdominal em adultos na cidade de Salvador, Bahia, Brasil. Cad Saúde Pública. 2009;25:570-82.

7. Barbosa JB, Silva AAM, Santos AM, Monteiro Júnior FC, Barbosa MM, Barbosa MM, et al. Prevalência da hipertensão arterial em adultos e fatores associados em São Luís - MA. Arq Bras Cardiol. 2008;91:260-6.

8. Barbosa JM, Cabral PC, Lira PIC, Florêncio TMMT. Fatores socioeconômicos associados ao excesso de peso em população de baixa renda do Nordeste brasileiro. Arch Latinoam Nutr. 2009;59:22-9.

9. Veloso HJF, Silva AAM. Prevalência e fatores associados à obesidade abdominal e ao excesso de peso em adultos maranhenses. Rev Bras Epidemiol. 2010;13:400-12.

10. Souza NP, Cesse EÂP, Souza WV, Fontbonne A, Barreto MNSC, Le Goff M, et al. Temporal variation in prevalence, awareness and control of hypertension in urban and rural areas in Northeast Brazil between 2006 and 2016. Cad Saúde Pública. 2020;36.

11. Correia LL, Silveira DMI, Silva AC, Campos JS, Machado MMT, Rocha HAL, et al. Prevalência e determinantes de obesidade e sobrepeso em mulheres em idade reprodutiva residentes na região semiárida do Brasil. Cienc Saúde Colet. 2011;16:133-45.

12. Ataíde Lima RP, de Carvalho Pereira D, Pordeus Luna RC, Gonçalves MCR, de Lima RT, Filho MB, et al. BMI, overweight status and obesity adjusted by various factors in all age groups in the population of a city in Northeastern Brazil. Int J Environ Res Public Health. 2015;12(4):4422-38.

13. Holanda LGM, Martins MCC, Souza Filho MD, Carvalho CMRG, Assis RC, Leal LMM, et al. Excesso de peso e adiposidade central em adultos de Teresina-PI. Rev Assoc Med Bras. 2011;57:50-5.

14. Soares D, Kochergin C. Fatores associados à obesidade em idosos quilombolas, Bahia, Brasil. Rev APS. 2018;20.

15. Santiago ERC, Diniz AS, Oliveira JS, Leal VS, Andrade MIS, Lira PIC. Prevalence of Systemic Arterial Hypertension and Associated Factors Among Adults from the Semi-Arid Region of Pernambuco, Brazil. Arq Bras Cardiol. 2019;113:687-95.

16. Mussi RFF, Petróski EL. Síndrome metabólica e fatores associados em quilombolas baianos, Brasil. Ciênc Saúde Colet. 2019;24:2481-90.

17. Lavôr LCDC, Sousa RRD, Rodrigues LARL, Rodrigues Filho ODS, Paiva ADA, Frota KDMG. Prevalence of arterial hypertension and associated factors: a population-based study. Revista da Associação Médica Brasileira. 2020;66(5):630-6.

18. Lolio CA, Latorre MRDO. Prevalência de obesidade em localidade do Estado de São Paulo, Brasil, 1987. Rev Saúde Pública. 1991;25:33-6.

19. Martins IS, Velásquez-Meléndez G, Cervato AM. Estado nutricional de grupamentos sociais da área metropolitana de São Paulo, Brasil. Cad Saúde Pública. 1999;15:41-78.

20. Bloch KV, Klein CH, Souza e Silva NA, Nogueira Ada R, Campos LH. Arterial hypertension and obesity in Ilha do Governador-Rio de Janeiro. Arq Bras Cardiol. 1994;62(1):17-22.

21. Ramos de Marins VM, Varnier Almeida RM, Pereira RA, Barros MB. Factors associated with overweight and central body fat in the city of Rio de Janeiro: results of a two-stage random sampling survey. Public Health. 2001;115(3):236-42.

22. Beleigoli AM, Boersma E, F. DM, Lima-Costa MF, Ribeiro AL. Overweight and class I obesity are associated with lower 10-year risk of mortality in Brazilian older adults: the Bambui Cohort Study of Ageing. PLoS One. 2012;7(12):e52111.

23. Lelis DF, Pereira AC, Krieger JE, Mill JG, Santos SHS, Baldo MP. Polymorphisms of the renin-angiotensin system are not associated with overweight and obesity in a general adult population. Arch Endocrinol Metab. 2019;63:402-10.

24. Barbosa AR, Souza JMP, Lebrão ML, Marucci MFN. Estado nutricional e desempenho motor de idosos de São Paulo. Rev Assoc Méd Bras. 2007;53:75-9.

25. Zaitune MPA, Barros MBA, César CLG, Carandina L, Goldbaum M. Hipertensão arterial em idosos: prevalência, fatores associados e práticas de controle no Município de Campinas, São Paulo, Brasil. Cad Saúde Pública. 2006;22:285-94.

26. Freitas SN, Caiaffa WT, César CC, Faria VA, Nascimento RM, Coelho GLLM. Risco nutricional na população urbana de Ouro Preto, sudeste do Brasil: estudo de corações de Ouro Preto. Arq Bras Cardiol. 2007;88:191-9.

27. Souza LJ, Gicovate Neto C, Chalita FEB, Reis AFF, Bastos DA, Souto Filho JTD, et al. Prevalência de obesidade e fatores de risco cardiovascular em Campos, Rio de Janeiro. Arq Bras Endocrinol Metab. 2003;47:669-76.

28. Bueno MB, Cesar CL, Martini LA, Fisberg RM. Dietary calcium intake and overweight: an epidemiologic view. Nutrition. 2008;24(11-12):1110-5.

29. Marcopito LF, Rodrigues SSF, Pacheco MA, Shirassu MM, Goldfeder AJ, Moraes MA. Prevalência de alguns fatores de risco para doenças crônicas na cidade de São Paulo. Rev Saúde Pública. 2005;39:738-45.

30. Bossan FM, Anjos LA, Vasconcellos MTL, Wahrlich V. Nutritional status of the adult population in Niterói, Rio de Janeiro, Brazil: the Nutrition, Physical Activity, and Health Survey. Cad Saúde Pública. 2007;23:1867-76.

31. Fiório CE, Cesar CLG, Alves MCGP, Goldbaum M. Prevalência de hipertensão arterial em adultos no município de São Paulo e fatores associados. Rev Bras Epidemiol. 2020;23.

32. Pimenta AM, Gazzinelli A, Velásquez-Meléndez G. Prevalência da síndrome metabólica e seus fatores associados em área rural de Minas Gerais (MG, Brasil). Cienc Saúde Coletiva. 2011;16:3297-306.

33. Gimeno SGA, Mondini L, Moraes SA, Freitas ICM. Padrões de consumo de alimentos e fatores associados em adultos de Ribeirão Preto, São Paulo, Brasil: Projeto OBEDIARP. Cad Saúde Pública. 2011;27:533-45.

34. Zangirolani L, Nucci L, Mafra A, Stephan C, Medeiros MA, Silveir L, et al. Overweight distribution and spatial risk for binomial response 2010.

35. Zangirolani LTO, Assumpção D, Medeiros MAT, Barros MBA. Hipertensão arterial autorreferida em adultos residentes em Campinas, São Paulo, Brasil: prevalência, fatores associados e práticas de controle em estudo de base populacional. Cien Saúde Colet. 2018;23:1221-32.

36. Borim FSA, Barros MBA, Neri AL. Autoavaliação da saúde em idosos: pesquisa de base populacional no Município de Campinas, São Paulo, Brasil. Cad Saúde Pública. 2012;28:769-80.

37. Fontes AS, Pallottini AC, Vieira DAS, Fontanelli MM, Marchioni DM, Cesar CLG, et al. Demographic, socioeconomic and lifestyle factors associated with sugar-sweetened beverage intake: a population-based study. Rev BrasEpidemiol. 2020;23.

38. Andrade RG, Chaves OC, Costa DAS, Andrade ACS, Bispo S, Felicissimo MF, et al. Overweight in men and women among urban area residents: individual factors and socioeconomic context. Cad Saúde Pública. 2015;31:148-58.

39. Canaan Rezende FA, Queiroz Ribeiro A, Priore SE, Castro Franceschini Sdo C. Anthropometric differences related to genders and age in the elderly. Nutr Hosp. 2015;32(2):757-64.

40. Martins TCR, Chagas RB, Andrade JdFR, Mendes DC, Souza LPS, Dias OV, et al. Exceso de peso y factores asociados: un estudio de base poblacional. Enferm Glob. 2016;15:51-62.

41. Nascimento MAS, Zucolotto DCC, Sartorelli DS. Associação entre a percepção de atributos ambientais e excesso de peso: um estudo realizado em um município de pequeno porte. Cad Saúde Pública. 2015;31:173-82.

42. Machado VS, Valadares AL, da Costa-Paiva LS, de Souza MH, Osis MJ, Pinto-Neto AM. Factors associated with self-perception of health among Brazilian women 50 years or older: a population-based study. Menopause. 2013;20(10):1055-60.

43. Silva FMO, Novaes TG, Ribeiro AQ, Longo GZ, Pessoa MC. Fatores ambientais associados à obesidade em população adulta de um município brasileiro de médio porte. Cad Saúde Pública. 2019;35.

44. Santos ÁS, Meneguci J, Scatena LM, Farinelli MR, Sousa MC, Damião R. Estudo de base populacional: perfil sociodemográfico e de saúde em idosos [Population-based study: socio-demographic and health profile of older adults] [Estudio de base poblacional: perfil sociodemográfico y de salud en ancianos]. Revista Enfermagem UERJ. 2018;26:e21473.

45. Matozinhos F, Gomes C, Mendes L, Pessoa M, Padez C, Pena G, et al. Association between the perceived environment and overweight in adults and elderly: a cross-sectional study. Nutrire. 2016;41.

46. Silva PAB, Sacramento ADJ, Carmo CIDDD, Silva LB, Silqueira SMDF, Soares SM. Factors associated with metabolic syndrome in older adults: a population-based study. Rev Bras Enferm. 2019;72(suppl 2):221-8.

47. Ferriani LO, Coutinho ESF, Silva DA, Faria CPD, Molina MDCB, Benseñor IJM, et al. Subestimativa de obesidade e sobrepeso a partir de medidas autorrelatadas na população geral: prevalência e proposta de modelos para correção. Cadernos de Saúde Pública. 2019;35(6).

48. Fuchs F, Moreira L, Moraes R, Bredemeier M, Cardozo S. Prevalência de Hipertensão Arterial Sistêmica e Fatores Associados na Região Urbana de Porto Alegre. Estudo de Base Populacional. Arq Bras Cardiol. 1994;63.

49. Piccini RX. Obesidade: constituição, atividade ou educação? Rev Ass Méd Bras. 1994;42:79-83.

50. Gigante DP, Barros FC, Post CLA, Olinto MTA. Prevalência de obesidade em adultos e seus fatores de risco. Rev Saúde Pública. 1997;31:236-46.

51. Bastos CA, Oppermann K, Fuchs SC, Donato GB, Spritzer PM. Determinants of ovarian volume in pre-, menopausal transition, and post-menopausal women: a population-based study. Maturitas. 2006;53(4):405-12.

52. Silveira EA, Kac G, Barbosa LS. Prevalência e fatores associados à obesidade em idosos residentes em Pelotas, Rio Grande do Sul, Brasil: classificação da obesidade segundo dois pontos de corte do índice de massa corporal. Cad Saúde Pública. 2009;25:1569-77.

53. Gus I, Fischmann A, Medina C. Prevalence of Risk Factors for Coronary Artery Disease in the Brazilian State of Rio Grande do Sul. Arq Bras Cardiol. 2002;78:484-90.

54. Olinto MTA, Nácul LC, Dias-da-Costa JS, Gigante DP, Menezes AMB, Macedo S. Níveis de intervenção para obesidade abdominal: prevalência e fatores associados. Cad Saúde Pública. 2006;22:1207-15.

55. Castanheira M, Olinto MTA, Gigante DP. Associação de variáveis sócio-demográficas e comportamentais com a gordura abdominal em adultos: estudo de base populacional no Sul do Brasil. Cad Saúde Pública. 2003;19:S55-S65.

56. Oliveira SS, Santos IdS, Silva JFP, Machado EC. Prevalência e fatores associados à doença do refluxo gastroesofágico. Arq Gastroenterol. 2005;42:116-21.

57. Perozzo G, Olinto MTA, Dias-da-Costa JS, Henn RL, Sarriera J, Pattussi MP. Associação dos padrões alimentares com obesidade geral e abdominal em mulheres residentes no Sul do Brasil. Cad Saúde Pública. 2008;24:2427-39.

58. Hallal PC, Reichert FF, Siqueira FV, Dumith SC, Bastos JP, da Silva MC, et al. Correlates of leisure-time physical activity differ by body-mass-index status in Brazilian adults. J Phys Act Health. 2008;5(4):571-8.

59. Sarturi JB, Neves J, Peres KG. Obesidade em adultos: estudo de base populacional num município de pequeno porte no sul do Brasil em 2005. Ciênc Saúde Colet. 2010;15:105-13.

60. Knuth AG, Bielemann RM, Silva SG, Borges TT, Del Duca GF, Kremer MM, et al. Conhecimento de adultos sobre o papel da atividade física na prevenção e tratamento de diabetes e hipertensão: estudo de base populacional no Sul do Brasil. Cad Saúde Pública. 2009;25:513-20.

61. Backes V, Olinto MTA, Henn RL, Cremonese C, Pattussi MP. Associação entre aspectos psicossociais e excesso de peso referido em adultos de um município de médio porte do Sul do Brasil. Cad Saúde Pública. 2011;27:573-80.

62. Fuchs SC, Moreira LB, Camey SA, Moreira MB, Fuchs FD. Clustering of risk factors for cardiovascular disease among women in Southern Brazil: a population-based study. Cad Saúde Pública. 2008;24:s285-s93.

63. Vedana EHB, Peres MA, Neves J, Rocha GC, Longo GZ. Prevalência de obesidade e fatores potencialmente causais em adultos em região do sul do Brasil. Arq Bras Endocrinol Metab. 2008;52:1156-62.

64. Silva DAS, Petroski EL, Peres MA. Pré-hipertensão e hipertensão em adultos de Florianópolis: estudo de base populacional. Rev Saúde Pública. 2012;46:988-98.

65. Goes VF, Wazlawik E, D'Orsi E, Gonzalez-Chica DA. Severe obesity increases the prevalence but not the incidence of depressive symptoms in the elderly-population-based cohort in Southern Brazil. Int Psychogeriatr. 2017;29(8):1307-16.

66. Dell Agnolo C, Gravena A, Lope T, Rocha-Brischiliari S, Carvalh M, Pelloso S, et al. Excesso de peso e fatores associados: estudo populacional no Sul do Brasil. Revista Brasileira de Pesquisa em Saúde. 2014;16:38-47.

67. Gravena AA, Brischiliari SC, Lopes TC, Agnolo CM, Carvalho MD, Pelloso SM. Excess weight and abdominal obesity in postmenopausal Brazilian women: a population-based study. BMC Womens Health. 2013;13:46.

68. Branco JC, Motta J, Wiener C, Oses JP, Pedrotti Moreira F, Spessato B, et al. Association between obesity and suicide in woman, but not in man: a population-based study of young adults. Psychol Health Med. 2017;22(3):275-81.

69. Linhares RS, Horta BL, Gigante DP, Dias-da-Costa JS, Olinto MTA. Distribuição de obesidade geral e abdominal em adultos de uma cidade no Sul do Brasil. Cad Saúde Pública. 2012;28:438-47.

70. Souza RKT, Bortoletto MSS, Loch MR, González AD, Matsuo T, Cabrera MAS, et al. Prevalência de fatores de risco cardiovascular em pessoas com 40 anos ou mais de idade, em Cambé, Paraná (2011): estudo de base populacional. Epidemiol Serv Saúde. 2013;22:435-44.

71. Souza ECC, Pizzichini MMM, Dias M, Cunha MJ, Matte DL, Karloh M, et al. Body mass index, asthma, and respiratory symptoms: a population-based study. J Bras Pneumol. 2020;46.

72. Cavalcanti AM, Kusma S, Chomatas ER, Ignácio SA, Mendes E, Moysés S, et al. Noncommunicable diseases and their common risk factors in Curitiba, Brazil: results of a cross-sectional, population-based study. Rev Panam Salud Publica. 2018:1-10.

73. Gus I, Ribeiro RA, Kato S, Bastos J, Medina C, Zazlavsky C, et al. Variations in the Prevalence of Risk Factors for Coronary Artery Disease in Rio Grande do Sul-Brazil: A Comparative Analysis between 2002 and 2014. Arq Bras Cardiol. 2015;105:573-9.

74. Costa CS, Schneider BC, Cesar JA. Obesidade geral e abdominal em idosos do Sul do Brasil: resultados do estudo COMO VAI? Ciênc Saúde Coletiva. 2016;21:3585-96.

75. Backes V, Bairros F, Cafruni CB, Cummins S, Shareck M, Mason K, et al. Food environment, income and obesity: a multilevel analysis of a reality of women in Southern Brazil. Cad Saúde Pública. 2019;35.

76. Xavier MO, Del-Ponte B, Santos IS. Epidemiology of smoking in the rural area of a medium-sized city in Southern Brazil. Rev Saúde Pública. 2018;52.

77. Dumith SC, Maciel FV, Borchardt JL, Alam VS, Silveira FC, Paulitsch RG. Preditores e condições de saúde associados à prática de atividade física moderada e vigorosa em adultos e idosos no sul do Brasil. Rev Bras Epidemiol. 2019;22.

78. Peixoto MdRG, Benício MHDA, Jardim PCBV. The relationship between body mass index and lifestyle in a Brazilian adult population: a cross-sectional survey. Cad Saúde Pública. 2007;23:2694-740.

79. Nascente FMN, Jardim PCBV, Peixoto MdRG, Monego ET, Barroso WKS, Moreira HG, et al. Hipertensão arterial e sua associação com índices antropométricos em adultos de uma cidade de pequeno porte do interior do Brasil. Rev Assoc Méd Bras. 2009;55:716-22.

80. Carnelosso ML, Barbosa MA, Porto CC, Silva SAe, Carvalho MMd, Oliveira ALI. Prevalência de fatores de risco para doenças cardiovasculares na região leste de Goiânia (GO). Ciênc Saúde Colet. 2010;15:1073-80.

81. Silva EC, Martins MSAS, Guimarães LV, Segri NJ, Lopes MAL, Espinosa MM. Prevalência de hipertensão arterial sistêmica e fatores associados em homens e mulheres residentes em municípios da Amazônia Legal. Rev Bras Epidemiol. 2016;19:38-51.

82. Sousa A, Da Costa T. Assessment of Nutrient and Food Group Intakes across Sex, Physical Activity, and Body Mass Index in an Urban Brazilian Population. Nutrients. 2018;10(11):1714.

83. Sichieri R, Coitinho DC, Leao MM, Recine E, Everhart JE. High temporal, geographic, and income variation in body mass index among adults in Brazil. Am J Public Health. 1994;84(5):793-8.

84. IBGE. Instituto Brasileiro de Geografia e Estatística. Pesquisa sobre padrões de vida 1996-1997 / IBGE, Departamento de População e Indicadores Sociais. Rio de Janeiro1999. 149 p.

85. Mendez MA, Monteiro CA, Popkin BM. Overweight exceeds underweight among women in most developing countries. Am J Clin Nutr. 2005;81(3):714-21.

86. Costa LC, Thuler LCS. Fatores associados ao risco para doenças não transmissíveis em adultos brasileiros: estudo transversal de base populacional. Rev Bras Estud Popul. 2012;29:133-45.

87. IBGE. Instituto Brasileiro de Geografia e Estatística. Pesquisa de orçamentos familiares 2002-2003: aquisição alimentar domiciliar per capita; Brasil e grandes regiões e unidades da Federação. Rio de Janeiro: IBGE; 2004.

88. Brasil. Ministerio da Saude. Pesquisa Nacional de Demografia e Saúde da Criança e da Mulher – PNDS 2006 : dimensões do processo reprodutivo e da saúde da criança/ Ministério da Saúde, Centro Brasileiro de Análise e Planejamento. Brasilia: Ministerio da Saude; 2009.

89. Corrêa MM, Tomasi E, Thumé E, Oliveira ERA, Facchini LA. Razão cintura-estatura como marcador antropométrico de excesso de peso em idosos brasileiros. Cad Saúde Pública. 2017;33.

90. Pavão ALB, Werneck GL, Campos MR. Autoavaliação do estado de saúde e a associação com fatores sociodemográficos, hábitos de vida e morbidade na população: um inquérito nacional. Cad Saúde Pública. 2013;29:723-34.

91. IBGE. Instituto Brasileiro de Geografia e Estatística. Pesquisa de orçamentos familiares 2008-2009 : antropometria e estado nutricional de crianças, adolescentes e adultos no Brasil / IBGE, Coordenação de trabalho e Rendimento. Rio de Janeiro2011. 127 p.

92. Ferriolli E, Pessanha F, Moreira VG, Dias RC, Neri AL, Lourenco RA. Body composition and frailty profiles in Brazilian older people: Frailty in Brazilian Older People Study-FIBRA-BR. Arch Gerontol Geriatr. 2017;71:99-104.

93. Ferreira A, Szwarcwald C, Damacena G. Prevalência e fatores associados da obesidade na população brasileira: estudo com dados aferidos da Pesquisa Nacional de Saúde, 2013. Rev Bras Epidemiol. 2019;22.

94. Firmo JOA, Mambrini JVM, Peixoto SV, Loyola Filho AI, Souza Junior PRB, Andrade FB, et al. Adequate control of hypertension among older adults: ELSI-Brazil. Rev Saúde Pública. 2018;52.

95. IBGE. Instituto Brasileiro de Geografia e Estatística. Pesquisa Nacional de Saúde 2019 - Microdados 2020 [Available from: <https://www.ibge.gov.br/estatisticas/downloads-estatisticas.html?caminho=PNS/2019/Microdados/Dados>.
