# Supplementary material for: Time-trend in excess weight in Brazilian adults: A systematic review and meta-analysis
Source: PLoS One. 2021 Sep 28;16(9):e0257755. doi: 10.1371/journal.pone.0257755 (PMC8478247; doi:10.1371/journal.pone.0257755)
Supplement: S1 Table — (DOCX) [file pone.0257755.s004.docx]

Supporting Information

Title: Time trend in excess weight in Brazilian adults: a systematic review and meta-analysis

Journal: Plos One

This supporting material presents the table of data extracted from the studies included in the systematic review and meta-analysis and their references. It also presents the funnel plots of the prevalence of excess weight, overweight and obesity in Brazilian adults, between 1974 and 2020

**S1 Table.** Electronic search strategy.

| **Database** | **Search strategy** |
| --- | --- |
| **PUBMED** | (overweight OR obesity OR (excess weight)) AND (brazil OR brasil) AND (prevalence OR prevalences) |
| **Embase** | #9. #7 AND #8 1,219 8 Jun 2021  #8. embase NOT medline 9,584,253 8 Jun 2021  #7. #1 AND #5 AND #6 5,060 8 Jun 2021  #6. 'brazil' 691,013 8 Jun 2021  #5. #2 OR #3 OR #4 683,180 8 Jun 2021  #4. 'excess weight' 9,782 8 Jun 2021  #3. 'overweight'/exp OR overweight 585,468 8 Jun 2021  #2. 'obesity'/exp OR obesity 673,202 8 Jun 2021  #1. 'prevalence'/exp OR prevalence 1,169,938 8 Jun 2021 |
| **Scopus** | ( TITLE-ABS-KEY ( *overweight* ) OR TITLE-ABS-KEY ( *obesity* ) OR TITLE-ABS-KEY ( *excess* AND *weight* ) ) AND ( TITLE-ABS-KEY ( *brazil* ) OR TITLE-ABS-KEY ( *brasil* ) ) AND ( TITLE-ABS-KEY ( *prevalence* ) OR TITLE-ABS-KEY ( *prevalences* ) ) AND NOT ( INDEX ( *medline* ) OR INDEX ( *embase* ) ) |
| **LILACS** | (obesity OR overweight OR (excess weight)) AND (prevalence) AND (brazil) AND (db:("LILACS") |
