# Supplementary material for: Time-trend in excess weight in Brazilian adults: A systematic review and meta-analysis
Source: PLoS One. 2021 Sep 28;16(9):e0257755. doi: 10.1371/journal.pone.0257755 (PMC8478247; doi:10.1371/journal.pone.0257755)
Supplement: S2 Table — This is the S2 Table legend. M, measured. SR, self-reported. BMI, body mass index. BR, Brazil. WHO, World Health Organization. PAHO, Pan American Health Organization. ENDEF, Estudo Nacional de Despesa Familiar (National Survey on Household Expenses). PNSN, Pesquisa Nacional de Saúde e Nutrição (National Survey on Health and Nutrition). PNDS, Pesquisa Nacional de Demografia e Saúde (National Demography and Health Survey). PPV, Pesquisa sobre Padrões de Vida (Living Standards Survey). POF, Pesquisa de Orçamentos Familiares (Household Budget Survey). PDSD, Pesquisa Dimensões Sociais das Desigualdades (Social Dimensions of Inequalilies Survey). PNS, Pesquisa Nacional de Saúde (National Health Survey). ELSI, Estudo Longitudinal da Saúde dos Idosos (Brazilian Longitudinal Study of Aging). aWHO–overweight:BMI ≥ 25 and <30 kg/m2, obesity:BMI ≥ 30kg/m2; Lipschitz–underweight: BMI less than 22kg/m2, overweight: BMI more than 27kg/m2; PAHO–underweight: BMI ≤ 23 kg/m2, overweight: BMI ≥ 28 kg/m2 and < 30 kg/m2, obesity: BMI ≥ 30 kg/m2. bCritical appraisal according to The Joanna Briggs Institute Critical Appraisal Checklist for Studies Reporting Prevalence Data: 1. Was the sample frame appropriate to address the target population? 2. Were study participants sampled in an appropriate way? 3. Was the sample size adequate? 4. Were the study subjects and the setting described in detail? 5. Was the data analysis conducted with sufficient coverage of the identified sample? 6. Were valid methods used for the identification of the condition? 7. Was the condition measured in a standard, reliable way for all participants? 8. Was there appropriate statistical analysis? 9. Was the response rate adequate, and if not, was the low response rate managed appropriately?. (DOCX) [file pone.0257755.s005.docx]

**S2 Table.** Main characteristics and quality assessment of included studies in meta-analysis.

| Region | First author,year | Year of survey | city | Sample  size | %women | Age (range) | Data (M/SR) | BMI classification^a^ | Critical appraisal^b^ | | | | | | | | | |
| --- | --- | --- | --- | --- | --- | --- | --- | --- | --- | --- | --- | --- | --- | --- | --- | --- | --- | --- |
|  |  |  |  |  |  |  |  |  | 1 | 2 | 3 | 4 | 5 | 6 | 7 | 8 | 9 | Final Score |
| NORTH | Lino, 2011(1) | 2007 | Rio Branco | 1469 | 55.8 | ≥ 18 | M | WHO | 1 | 1 | 1 | 1 | 1 | 1 | 1 | 1 | 1 | 9 |
|  | Cordeiro, 2016(2) | 2015 | Tefé | 451 | 58.3 | ≥ 18 | M | WHO | 1 | 1 | 0 | 1 | 1 | 1 | 1 | 1 | 0 | 7 |
| NORTHEAST | Costa, 2014(3) | 1997 | Pernambuco (State) | 802 | 100 | 19-49 | M | WHO | 1 | 1 | 1 | 1 | 1 | 1 | 1 | 1 | 1 | 9 |
|  | Florencio, 2001(4) | 1999 | Maceio | 532 | 52.8 | ≥ 18 | M | WHO | 1 | 1 | 1 | 0 | 1 | 0 | 0 | 1 | 1 | 6 |
|  | Lessa, 2006(5) | 1999 | Salvador | 1439 | 57.7 | ≥ 20 | M | WHO | 1 | 1 | 1 | 1 | 1 | 1 | 1 | 1 | 1 | 9 |
|  | Oliveira, 2009(6) | 2001 | Salvador | 557 | 64.1 | 20-59 | M | WHO | 1 | 1 | 0 | 1 | 1 | 1 | 1 | 1 | 1 | 8 |
|  | Barbosa, 2008(7) | 2003 | São Luis | 835 | 59.3 | ≥ 18 | M | WHO | 1 | 1 | 0 | 1 | 1 | 1 | 1 | 1 | 0 | 7 |
|  | Barbosa, 2009(8) | 2004 | Maceio | 3214 | 63.8 | 20-69 | M | WHO | 1 | 1 | 1 | 1 | 1 | 1 | 0 | 0 | 1 | 7 |
|  | Veloso, 2010(9) | 2006 | Maranhão | 1005 | 60.9 | 20-59 | M | WHO | 1 | 1 | 1 | 1 | 1 | 1 | 1 | 1 | 1 | 9 |
|  | Souza, 2020a(10) | 2006 | Pernambuco (State) | 1502 | 63.3 | ≥ 20 | M | WHO | 1 | 1 | 1 | 1 | 1 | 1 | 1 | 1 | 1 | 9 |
|  | Correia, 2011(11) | 2007 | Ceará | 6845 | 100.0 | 20-49 | M | WHO | 1 | 1 | 1 | 1 | 1 | 1 | 1 | 1 | 0 | 8 |
|  | Ataide Lima, 2015(12) | 2008 | João Pessoa | 665 | 76.5 | ≥ 20 | M | WHO | 1 | 1 | 1 | 1 | 1 | 1 | 1 | 1 | 1 | 9 |
|  | Holanda, 2011(13) | 2010 | Teresina | 464 | 64.6 | 20-59 | M | WHO | 1 | 1 | 0 | 1 | 1 | 1 | 1 | 0 | 0 | 6 |
|  | Soares, 2017(14) | 2011 | Vitoria da Conquista | 177 | 51.4 | ≥ 60 | M | WHO | 1 | 1 | 0 | 1 | 1 | 1 | 1 | 1 | 1 | 8 |
|  | Santiago, 2019(15) | 2015 | Sertão Pernambuco | 416 | 64.9 | 20-59 | M | WHO | 1 | 1 | 0 | 1 | 1 | 1 | 1 | 1 | 1 | 8 |
|  | Souza, 2020b(10) | 2015 | Pernambuco (State) | 1053 | 60.8 | ≥ 20 | M | WHO | 1 | 1 | 1 | 1 | 1 | 1 | 1 | 1 | 1 | 9 |
|  | Mussi, 2019(16) | 2016 | Guanambi | 850 | 60.2 | ≥ 18 | M | WHO | 1 | 1 | 0 | 1 | 1 | 1 | 1 | 1 | 1 | 8 |
|  | Lavôr, 2020(17) | 2018 | Teresina/Picos | 1057 |  | 20-59 | M | WHO | 1 | 1 | 1 | 1 | 1 | 1 | 1 | 1 | 1 | 9 |
| SOUTHEAST | Lolio, 1991(18) | 1987 | Araraquara | 1126 | 55.4 | 18-74 | M | WHO | 1 | 1 | 1 | 1 | 1 | 1 | 1 | 0 | 1 | 8 |
|  | Martins, 1999(19) | 1990 | Cotia | 1041 | 58.9 | ≥ 20 | M | WHO | 1 | 1 | 1 | 1 | 1 | 1 | 1 | 1 | 1 | 9 |
|  | Bloch, 1994(20) | 1991 | Ilha do Governador | 1268 | - | ≥ 20 | M | WHO | 1 | 1 | 1 | 1 | 1 | 1 | 1 | 1 | 0 | 8 |
|  | Ramos de Marins, 2001(21) | 1995 | Rio de Janeiro | 3252 | 56.5 | ≥ 20 | M | WHO | 1 | 1 | 1 | 1 | 1 | 1 | 1 | 1 | 1 | 9 |
|  | Beleigoli, 2012(22) | 1997 | Bambui | 1450 | 60.9 | ≥ 60 | M | WHO | 1 | 1 | 1 | 1 | 1 | 1 | 1 | 1 | 1 | 9 |
|  | Lelis, 2019(23) | 1999 | Vitória | 1567 | 54.4 | 25-64 | M | WHO | 1 | 1 | 1 | 1 | 1 | 1 | 1 | 1 | 1 | 9 |
|  | Barbosa, 2007(24) | 2000 | São Paulo | 1796 | 59.2 | ≥ 60 | M | Lipschitz | 1 | 1 | 1 | 1 | 1 | 1 | 1 | 1 | 1 | 9 |
|  | Zaitune, 2006(25) | 2001 | Campinas | 426 | 48.8 | ≥ 60 | SR | WHO | 1 | 1 | 0 | 1 | 1 | 0 | 0 | 1 | 1 | 6 |
|  | Freitas, 2007(26) | 2001 | Ouro Preto | 768 | 50.8 | ≥ 15 | M | WHO | 1 | 1 | 0 | 1 | 1 | 1 | 1 | 1 | 1 | 8 |
|  | Souza, 2003(27) | 2001 | Campos | 1039 | 52.2 | ≥ 18 | M | WHO | 1 | 1 | 1 | 1 | 1 | 1 | 1 | 1 | 1 | 9 |
|  | Bueno, 2008(28) | 2001 | São Paulo (State) | 1459 | 50.2 | 20-59 | SR | WHO | 1 | 1 | 1 | 1 | 1 | 0 | 0 | 1 | 1 | 7 |
|  | Marcopito, 2005(29) | 2001 | São Paulo | 2103 | 53.5 | 15-59 | M | WHO | 1 | 1 | 1 | 1 | 1 | 1 | 1 | 1 | 1 | 9 |
|  | Bossan, 2007(30) | 2003 | Niteroi | 3096 | 62.7 | ≥ 20 | M | WHO | 1 | 1 | 1 | 1 | 1 | 1 | 1 | 1 | 1 | 9 |

continues

**S2 Table.** *Continued*

| Region | First author,year | Year of survey | city | Sample  size | %women | Age (range) | Data (M/SR) | BMI classification^a^ | Critical appraisal^b^ | | | | | | | | | |
| --- | --- | --- | --- | --- | --- | --- | --- | --- | --- | --- | --- | --- | --- | --- | --- | --- | --- | --- |
|  |  |  |  |  |  |  |  |  | 1 | 2 | 3 | 4 | 5 | 6 | 7 | 8 | 9 | Final Score |
| SOUTHEAST (cont.) | Fiorio, 2020a(31) | 2003 | São Paulo | 1667 | - | ≥ 20 | SR | WHO | 1 | 1 | 1 | 1 | 1 | 1 | 0 | 1 | 1 | 8 |
|  | Pimenta, 2011(32) | 2004 | Vale do Jequitinhonha | 534 | 49.4 | ≥ 18 | M | WHO | 1 | 1 | 0 | 1 | 1 | 1 | 1 | 1 | 1 | 8 |
|  | Gimeno, 2011(33) | 2005 | Ribeirão Preto | 930 | 70.2 | ≥ 30 | M | WHO | 1 | 1 | 1 | 1 | 1 | 1 | 1 | 1 | 1 | 9 |
|  | Zangirolani,2010(34) | 2006 | Campinas | 651 | 55.8 | 19-65 | M | WHO | 1 | 1 | 0 | 1 | 1 | 1 | 1 | 1 | 1 | 8 |
|  | Zangirolani, 2018(35) | 2008 | Campinas | 937 | 54.2 | 20-59 | SR | WHO | 1 | 1 | 0 | 1 | 1 | 1 | 0 | 1 | 1 | 7 |
|  | Borim, 2012(36) | 2008 | Campinas | 1383 | 57.2 | ≥ 60 | SR | Lipschitz | 1 | 1 | 1 | 1 | 1 | 0 | 0 | 1 | 1 | 7 |
|  | Fontes, 2020(37) | 2008 | São Paulo | 2086 | 55.9 | ≥ 20 | SR | WHO | 1 | 1 | 1 | 1 | 1 | 1 | 0 | 1 | 1 | 8 |
|  | Andrade, 2015(38) | 2008 | Belo Horizonte | 2935 | 58.4 | 20-60 | M | WHO | 1 | 1 | 1 | 1 | 1 | 1 | 1 | 1 | 1 | 9 |
|  | Canaan Rezende,2015(39) | 2009 | Viçosa | 551 | 53.3 | ≥ 60 | M | Lischitz | 1 | 1 | 0 | 1 | 1 | 1 | 1 | 0 | 1 | 7 |
|  | Martins, 2016(40) | 2010 | Montes Claros | 2038 | 63.6 | ≥ 18 | M | WHO | 1 | 1 | 1 | 1 | 1 | 1 | 1 | 1 | 1 | 9 |
|  | Nascimento, 2015(41) | 2011 | Itirapuã | 216 | 55.0 | 18-60 | M | WHO | 1 | 1 | 0 | 1 | 1 | 1 | 1 | 1 | 0 | 7 |
|  | Machado, 2013(42) | 2011 | Campinas | 501 | 100.0 | ≥ 50 | SR | WHO | 1 | 1 | 0 | 1 | 1 | 0 | 0 | 1 | 1 | 6 |
|  | Silva, 2019(43) | 2012 | Viçosa | 965 | 55.2 | 20-59 | M | WHO | 1 | 1 | 0 | 1 | 1 | 1 | 1 | 1 | 1 | 8 |
|  | Santos, 2018(44) | 2012 | Uberaba | 3430 | 61.5 | ≥ 60 | M | WHO | 1 | 1 | 1 | 1 | 1 | 1 | 1 | 1 | 1 | 9 |
|  | Matozinhos, 2016(45) | 2013 | Montes Claros | 808 | 52.7 | ≥ 18 | M | WHO | 1 | 1 | 0 | 1 | 1 | 1 | 1 | 1 | 0 | 7 |
|  | Silva, 2019(46) | 2014 | Belo Horizonte | 271 | 67.5 | ≥ 60 | M | Lipschitz | 1 | 1 | 0 | 1 | 1 | 1 | 1 | 1 | 1 | 8 |
|  | Fiório, 2020b(31) | 2015 | São Paulo | 3184 | - | ≥ 20 | SR | WHO | 1 | 1 | 1 | 1 | 1 | 1 | 0 | 1 | 1 | 8 |
|  | Ferriani, 2019(47) | 2005 | São Paulo | 4151 | - | 18-60 | SR | WHO | 1 | 1 | 1 | 1 | 1 | 1 | 1 | 1 | 1 | 9 |
| SOUTH | Fuchs, 1994(48) | 1992 | Porto Alegre | 1091 | 55.1 | ≥ 18 | M | WHO | 1 | 1 | 1 | 1 | 1 | 1 | 1 | 1 | 1 | 9 |
|  | Piccini, 1994(49) | 1992 | Pelotas | 1657 | 56.4 | 20-69 | M | WHO | 1 | 1 | 1 | 1 | 1 | 1 | 1 | 1 | 1 | 9 |
|  | Gigante, 1997(50) | 1994 | Pelotas RS | 1035 | 56.0 | 20-69 | M | WHO | 1 | 1 | 1 | 1 | 1 | 1 | 1 | 1 | 1 | 9 |
|  | Bastos, 2006(51) | 1995 | Passo Fundo | 273 | 100.0 | 36-62 | M | WHO | 1 | 1 | 0 | 1 | 1 | 1 | 1 | 1 | 1 | 8 |
|  | Silveira, 2009(52) | 1999 | Pelotas RS | 596 | 59.4 | ≥ 60 | SR | WHO | 1 | 1 | 0 | 1 | 1 | 0 | 1 | 1 | 0 | 6 |
|  | Gus, 2002(53) | 1999 | Rio Grande do Sul (State) | 1051 | 51.9 | ≥ 20 | M | WHO | 1 | 1 | 1 | 1 | 1 | 0 | 0 | 1 | 1 | 7 |
|  | Olinto, 2006(54) | 1999 | Pelotas | 1935 | 56.7 | 20-69 | M | WHO | 1 | 1 | 1 | 1 | 1 | 1 | 1 | 1 | 1 | 9 |
|  | Castanheira, 2003(55) | 1999 | Pelotas | 3464 | 56.2 | 20-69 | SR | WHO | 1 | 1 | 1 | 1 | 1 | 1 | 1 | 1 | 1 | 9 |
|  | de Oliveira, 2005(56) | 1999 | Pelotas | 3934 | 57.0 | ≥ 20 | SR | WHO | 1 | 1 | 1 | 1 | 1 | 1 | 0 | 1 | 1 | 8 |
|  | Perozzo, 2008(57) | 2003 | São Leopoldo | 996 | 100.0 | 20-60 | M | WHO | 1 | 1 | 1 | 1 | 1 | 1 | 1 | 1 | 1 | 9 |
|  | Hallal, 2008(58) | 2003 | Pelotas | 3047 | 55.6 | ≥ 20 | SR | WHO | 1 | 1 | 1 | 1 | 1 | 0 | 0 | 1 | 1 | 7 |

continues

**S2 Table.** *Continued*

| Region | First author,year | Year of survey | city | Sample  size | %women | Age (range) | Data (M/SR) | BMI classification^a^ | Critical  appraisal^b^ | | | | | | | | | |
| --- | --- | --- | --- | --- | --- | --- | --- | --- | --- | --- | --- | --- | --- | --- | --- | --- | --- | --- |
|  |  |  |  |  |  |  |  |  | 1 | 2 | 3 | 4 | 5 | 6 | 7 | 8 | 9 | Final Score |
| SOUTH (cont) | Sarturi, 2010(59) | 2005 | Santo Ângelo | 434 | 58.3 | 20-59 | M | WHO | 1 | 1 | 0 | 1 | 1 | 1 | 1 | 1 | 1 | 8 |
|  | Knuth, 2009(60) | 2006 | Pelotas | 885 | 57.0 | 20-69 | SR | WHO | 1 | 1 | 0 | 1 | 1 | 0 | 0 | 1 | 1 | 6 |
|  | Backes, 2011(61) | 2007 | São Leopoldo | 1058 | 71.8 | ≥ 18 | SR | WHO | 1 | 1 | 1 | 1 | 1 | 0 | 0 | 1 | 1 | 7 |
|  | Fuchs, 2008(62) | 2007 | Porto Alegre | 1077 | 100.0 | 18-90 | M | WHO | 1 | 1 | 1 | 1 | 1 | 1 | 1 | 1 | 1 | 9 |
|  | Vedana, 2008(63) | 2007 | Lages | 1965 | 61.1 | 20-59 | M | WHO | 1 | 1 | 1 | 1 | 1 | 1 | 1 | 1 | 1 | 9 |
|  | Silva, 2012(64) | 2009 | Florianopolis | 1640 | 58.5 | 20-59 | M | WHO | 1 | 1 | 1 | 1 | 1 | 1 | 1 | 1 | 1 | 9 |
|  | Goes, 2017(65) | 2009 | Florianópolis | 1614 | 67.4 | ≥ 60 | M | WHO | 1 | 1 | 1 | 1 | 1 | 1 | 1 | 1 | 1 | 9 |
|  | DellAgnolo, 2013(66) | 2010 | Maringá | 369 | 74.0 | ≥ 18 | SR | WHO | 1 | 1 | 0 | 1 | 1 | 0 | 0 | 1 | 1 | 6 |
|  | Gravena, 2013(67) | 2010 | Maringá | 456 | 100.0 | 45-69 | M | WHO | 1 | 1 | 0 | 1 | 1 | 1 | 1 | 1 | 0 | 7 |
|  | Branco, 2017(68) | 2010 | Pelotas | 1953 | 54.9 | 18-35 | M | WHO | 1 | 0 | 1 | 0 | 0 | 1 | 0 | 1 | 0 | 4 |
|  | Linhares, 2012(69) | 2010 | Pelotas | 2448 | 58.3 | ≥ 20 | M | WHO | 1 | 1 | 1 | 1 | 1 | 1 | 1 | 1 | 1 | 9 |
|  | Souza, 2013(70) | 2011 | Cambé | 1180 | 54.4 | ≥ 40 | M | WHO | 1 | 1 | 1 | 1 | 1 | 1 | 1 | 1 | 1 | 9 |
|  | Souza, 2020(71) | 2012 | Florianópolis | 1026 | 59.6 | ≥ 40 | M | WHO | 1 | 1 | 1 | 1 | 1 | 1 | 1 | 1 | 1 | 9 |
|  | Cavalcanti, 2018(72) | 2013 | Curitiba | 1103 | 53.3 | ≥ 18 | M | WHO | 1 | 1 | 1 | 1 | 1 | 1 | 1 | 1 | 1 | 9 |
|  | Gus,,2015(73) | 2014 | Rio Grande do Sul (State) | 1059 | 56.6 | ≥ 18 | M | WHO | 1 | 1 | 1 | 1 | 1 | 0 | 0 | 1 | 1 | 7 |
|  | Costa, 2016(74) | 2014 | Pelotas | 1364 | 63.0 | ≥ 60 | M | WHO | 1 | 1 | 1 | 1 | 1 | 1 | 1 | 1 | 1 | 9 |
|  | Backes, 2019(75) | 2015 | São Leopoldo | 1096 | 100.0 | 20-69 | SR | WHO | 1 | 1 | 1 | 1 | 1 | 1 | 0 | 1 | 1 | 8 |
|  | Xavier, 2018(76) | 2016 | Pelotas | 1433 | 54.8 | ≥ 18 | M | WHO | 1 | 1 | 1 | 1 | 1 | 1 | 1 | 1 | 1 | 9 |
|  | Dumith, 2019(77) | 2016 | Rio Grande | 1290 | - | ≥ 18 | SR | WHO | 1 | 1 | 1 | 0 | 1 | 1 | 0 | 1 | 1 | 7 |
| MIDWEST | Peixoto, 2007(78) | 2001 | Goiânia | 1252 | 65.0 | 20-64 | M | WHO | 1 | 1 | 1 | 1 | 1 | 1 | 1 | 1 | 1 | 9 |
|  | Nascente, 2009(79) | 2002 | Firminópolis | 1168 | 63.2 | ≥ 18 | M | WHO | 1 | 1 | 1 | 1 | 1 | 1 | 1 | 1 | 1 | 9 |
|  | Carnelosso, 2010(80) | 2004 | Goiania | 3275 | 60.9 | ≥ 15 | M | WHO | 1 | 1 | 1 | 0 | 1 | 1 | 0 | 1 | 0 | 6 |
|  | Silva, 2016(81) | 2007 | Mato Grosso (State) | 1296 | 61.0 | 20-59 | M | WHO | 1 | 1 | 1 | 1 | 1 | 1 | 1 | 1 | 1 | 9 |
|  | Sousa, 2018(82) | 2016 | Brasilia (DF) | 506 |  | ≥ 20 | M | WHO | 1 | 1 | 0 | 1 | 1 | 0 | 0 | 1 | 1 | 6 |
| NATIONAL SURVEY | ENDEF, 1974(83) | 1974 | Brazil | 128307 | 50.8 | ≥ 18 | M | WHO | 1 | 1 | 1 | 1 | 1 | 1 | 1 | 1 | 1 | 9 |
|  | PNSN, 1989(83) | 1989 | Brazil | 33951 | 50.6 | ≥ 18 | M | WHO | 1 | 1 | 1 | 1 | 1 | 1 | 1 | 1 | 1 | 9 |
|  | PPV, 1996(84) | 1996 | Brazil | 10893 | 54.9 | ≥ 18 | M | WHO | 1 | 1 | 1 | 1 | 1 | 1 | 1 | 1 | 1 | 9 |
|  | PNDS, 1996(85) | 1996 | Brazil | 9251 | 100.0 | ≥ 18 | M | WHO | 1 | 1 | 1 | 1 | 1 | 1 | 1 | 1 | 1 | 9 |
|  | Costa, 2012(86) | 2002 | Brazil | 19252 | 57.1 | ≥ 18 | SR | WHO | 1 | 1 | 1 | 1 | 1 | 0 | 0 | 1 | 1 | 7 |
|  | POF, 2002(87) | 2002 | Brazil | 102457 | 51.4 | ≥ 18 | M | WHO | 1 | 1 | 1 | 1 | 1 | 1 | 1 | 1 | 1 | 9 |
|  | PNDS, 2006(88) | 2006 | Brazil | 13375 | 100.0 | ≥ 18 | M | WHO | 1 | 1 | 1 | 1 | 1 | 1 | 1 | 1 | 1 | 9 |
|  | Corrêa, 2017(89) | 2008 | Brazil | 5276 | 63.0 | ≥ 60 | M | WHO | 1 | 1 | 1 | 1 | 1 | 1 | 1 | 1 | 1 | 9 |
|  | PDSD, 2008(90) | 2008 | Brazil | 12056 | 58.1 | ≥ 18 | M | WHO | 1 | 1 | 1 | 1 | 1 | 1 | 1 | 1 | 1 | 9 |
|  | POF, 2008(91) | 2008 | Brazil | 104706 | 51.9 | ≥ 18 | M | WHO | 1 | 1 | 1 | 1 | 1 | 1 | 1 | 1 | 1 | 9 |
|  | Ferriolli, 2017(92) | 2009 | Brazil | 5638 | 66.2 | ≥ 65 | M | WHO | 1 | 1 | 1 | 1 | 1 | 1 | 1 | 1 | 1 | 9 |
|  | PNS, 2013(93) | 2013 | Brazil | 59402 | 56.4 | ≥ 18 | M | WHO | 1 | 1 | 1 | 1 | 1 | 1 | 1 | 1 | 1 | 9 |
|  | ELSI, 2015(94) | 2015 | Brazil | 8974 | 56.3 | ≥ 50 | M | PAHO | 1 | 1 | 1 | 1 | 1 | 1 | 1 | 1 | 1 | 9 |
|  | PNS, 2019(95) | 2019 | Brazil | 6730 | 49.6 | ≥ 18 | M | WHO | 1 | 1 | 1 | 1 | 1 | 1 | 1 | 1 | 1 | 9 |

This is the S2 Table legend.

M, measured. SR, self-reported. BMI, body mass index. BR, Brazil. WHO, World Health Organization. PAHO, Pan American Health Organization. ENDEF, Estudo Nacional de Despesa Familiar (National Survey on Household Expenses). PNSN, Pesquisa Nacional de Saúde e Nutrição (National Survey on Health and Nutrition). PNDS, Pesquisa Nacional de Demografia e Saúde (National Demography and Health Survey). PPV, Pesquisa sobre Padrões de Vida (Living Standards Survey). POF, Pesquisa de Orçamentos Familiares (Household Budget Survey). PDSD, Pesquisa Dimensões Sociais das Desigualdades (Social Dimensions of Inequalities Survey). PNS, Pesquisa Nacional de Saúde (National Health Survey). ELSI, Estudo Longitudinal da Saúde dos Idosos (Brazilian Longitudinal Study of Aging).

^a^WHO – overweight:BMI ≥ 25 and <30 kg/m^2^, obesity:BMI ≥ 30kg/m^2^; Lipschitz – underweight: BMI less than 22kg/m^2^, overweight: BMI more than 27kg/m^2^; PAHO – underweight: BMI ≤ 23 kg/m^2^, overweight: BMI ≥ 28 kg/m^2^ and < 30 kg/m2, obesity: BMI ≥ 30 kg/m^2^.

^b^Critical appraisal according to The Joanna Briggs Institute Critical Appraisal Checklist for Studies Reporting Prevalence Data:

1. Was the sample frame appropriate to address the target population?

2. Were study participants sampled in an appropriate way?

3. Was the sample size adequate?

4. Were the study subjects and the setting described in detail?

5. Was the data analysis conducted with sufficient coverage of the identified sample?

6. Were valid methods used for the identification of the condition?

7. Was the condition measured in a standard, reliable way for all participants?

8. Was there appropriate statistical analysis?

9. Was the response rate adequate, and if not, was the low response rate managed appropriately?
