# Supplementary material for: Time-trend in excess weight in Brazilian adults: A systematic review and meta-analysis
Source: PLoS One. 2021 Sep 28;16(9):e0257755. doi: 10.1371/journal.pone.0257755 (PMC8478247; doi:10.1371/journal.pone.0257755)
Supplement: Attachment — Submitted filename: Response_to_reviewers_2021_06.docx [file pone.0257755.s006.docx]

Response to reviewers

PONE-D-20-38212
Time-trend of excess weight in Brazil: a systematic review and meta-analysis.

Dear Editor,

Thank you for all the comments. We believe that the manuscript has been improved. Please check our response above

| Reviewer | Comments | Our response |
| --- | --- | --- |
| Editor | a. Please clarify the sources of funding (financial or material support) for your study. List the grants or organizations that supported your study, including funding received from your institution. b. State what role the funders took in the study. If the funders had no role in your study, please state: “The funders had no role in study design, data collection and analysis, decision to publish, or preparation of the manuscript.” c. If any authors received a salary from any of your funders, please state which authors and which funders. d. If you did not receive any funding for this study, please state: “The authors received no specific funding for this work.” | This study was financed in part by the Coordenação de Aperfeiçoamento de Pessoal de Nível Superior – CAPES (BRASIL) – Finace Code 001  b. “The funders had no role in study design, data collection and analysis, decision to publish, or preparation of the manuscript.” |
| Reviewer 1 | Title: • I suggest including the studied population in the title. | Time-trend in excess weight in Brazilian adults: a systematic review and meta-analysis. |
| Reviewer 1 | Introduction:  • I suggest bringing evidence-based studies which prove that there is no previous systematic review about the topic addressed. This information cannot be empirical; | Previous analysis only included national representative studies. Searching in the main databases doesn’t show studies that included local, regional, and national surveys. |
| Reviewer 1 | Introduction:  • Including epidemiological data about the excess of weight in Brazil; | We included this information in the text. |
| Reviewer 1 | Introduction:  • Evaluating the possibility of including in the “study objective” the period of time in which the temporal tendency was assessed. | We included this information in the text. |
| Reviewer 1 | Methodology:  • Were the researchers who assessed the eligibility criteria blindfolded? It is important to describe this; | Yes, the researchers were blindfolded. They used the Covidence platform and independently assessed all data. |
| Reviewer 1 | Methodology:  • Why was the adult population aged 18 or older considered? According to WHO criteria, for the assessment of nutritional status/growth curves, people aged between 10 and 19 years old are considered adolescents. I recommend justifying this choice of the methodology. | We considered according to the age of majority criterion in Brazil.  <http://www.planalto.gov.br/ccivil_03/leis/2002/L10406compilada.htm> |
| Reviewer 1 | Methodology:  • The article is registered in PROSPERO, but it is not up to date. I recommend reviewing it. | The register in PROSPERO was updated. |
| Reviewer 2 | Study overview  The field of the study is important and relevant since excess weight is a serious public health problem worldwide. However, the study has several methodological problems mainly in the Methods and Results sections. The justification for the study needs to be included in the introduction | We included this information in the text. |
| Reviewer 2 | Title:  1. The title should state that the study was carried out in adults: “Time trend in excess weight in Brazilian adults: a systematic review and meta-analysis.” | We accept the suggestion and change the title. |
| Reviewer 2 | Abstract:  2. At the end of the conclusion, the authors state: “Excess weight affected half of the adult Brazilian population, both sexes and in all regions of Brazil.”. However, they do not report the most prevalent sex and region in the Results section. | We performed new analyses considering sex and period of years. |
| Reviewer 2 | Abstract:  3. The authors reported that 86 studies and 8 national surveys were included in the study. However, the results are different from in Figure 1 “…(n = 91), that refers to 94 studies”? | We've updated Fig 1 according PRISMA-2020 Flow diagram and improved the designations for clarity (reports and studies).  <http://www.prisma-statement.org/PRISMAStatement/FlowDiagram> |
| Reviewer 2 | Introduction:  4. Page 3, line 59: Please add references. | We included this information in the text. |
| Reviewer 2 | Introduction:  5. Page 3, lines 64-65: “This review aimed to estimate the time-trend prevalence of excess weight in the Brazilian adult population.”. Please include the period investigated. | We included the period investigated. |
| Reviewer 2 | Introduction:  6. I was unable to locate the justification for the study. Why did the authors decide to do a systematic review on excess weight involving Brazilian adults? | Previous analysis only included national representative studies. Searching in the main databases doesn’t show studies that included local, regional, and national surveys. |
| Reviewer 2 | Methods:  7. The Methods section is poorly described. The authors should follow the PRISMA checklist to better describe the methods of the study. | We structured the text according to PRISMA checklist (2020). |
| Reviewer 2 | Methods:  8. Page 4, line 71-73: Did the authors include a grey literature search? The grey literature refers to unpublished research or published in non-commercial form and allows a more comprehensive search strategy. | We included this information in the text. As a strategy to identify any studies not indexed in databases, we searched in the references of the included studies. |
| Reviewer 2 | Methods:  9. Page 4, line 75: Did the authors consider longitudinal studies in the eligibility criteria? If so, it is important to mention. Otherwise justify. Also, were letters, personal opinions, book chapters or conference abstracts considered? | Yes, we considered longitudinal population-based studies that described the baseline of the study and not the segment.  Other sources were used as a strategy to identify additional studies. |
| Reviewer 2 | Methods:  10. Page 4, line 78: Were studies involving people with other comorbidities (diabetes, cardiovascular diseases, …) also excluded? This must be mentioned | We included all population-based studies regardless the presence of comorbidities. |
| Reviewer 2 | Methods:  11. Page 4, line 80: “…and children (< 18 years old)…” repeated from “b”, line 77. | We remove the information in duplicate. |
| Reviewer 2 | Methods:  12. Page 5, line 95: Include checklist reference. I suggest inserting the checklist as complementary material. | We included this information in the text. |
| Reviewer 2 | Methods:  13. Page 5, lines 99-101: “The total score obtained could range from zero (low quality) to nine (high quality). Surveys were considered adequate when they scored ≥ 6.” Please justify or include a reference explaining why the authors chose scored ≥ 6 as adequate. | The information was removed from the text. |
| Reviewer 2 | Methods:  14. S1 Table. Electronic search strategy: It is difficult to understand why the authors did not use other important key words in the search strategy, such as “excess weight” and “excess body weight”. In fact, the keyword “excess weight” appears in the title of this review! In a quick search using the terms “(excess weight OR excess body weight) AND (brazil OR brasil) AND (prevalence OR prevalences)”, I found 642 articles! | New searches were performed in the databases including the keyword “excess weight”. |
| Reviewer 2 | Methods:  15. Still regarding the search strategy, many studies report the prevalence of overweight/obesity/excess weight throughout the article and not in the title or abstract because it is not the main objective of the article. Did the authors consider this fact in the search? | Any mention of excess weight, overweight or obesity was considered to include the study in the full text review. |
| Reviewer 2 | Results:  16. The authors should follow the PRISMA checklist to better describe the results of the study. | We structured the text according to PRISMA checklist (2020). |
| Reviewer 2 | Results:  17. Fig 1: What do the authors mean by “Additional records through other sources”? This explanation was not mentioned in the Methods section. | We've updated Fig 1 according PRISMA-2020 Flow diagram and improved the designations for clarity (reports and studies). |
| Reviewer 2 | Results:  18. Page 6, line 124, S2 Table: I suggest including S2 Table as Table 1 in the Results section. Change the current Table 1 to Table 2. Qualitative data must be shown before meta-analysis data. | We believe that the extension of S2 Table can affect the presentation of the text. |
| Reviewer 2 | Results:  19. Page 6, line 129: “The prevalence …” of what? Excess weight? Overweigh? Please, define. The same inconsistency in line 131. | We included this information in the text. |
| Reviewer 2 | Results:  20. Page 6, lines 125, 127, 131-32, Table 1, and Fig 2: Which means, “excess weight”? The authors did not define this term before and did not consider it in the search strategy. | We included this information in the Methods section. |
| Reviewer 2 | Results:  21. Page 6, lines 132-34: “Between 1974 and 2004, the prevalence of excess weight was 40.2% (95% CI: 36.8; 43.7%) and from 2005 onwards, it has been 51.7% (95% CI: 134 48.8; 54.6%).” Does the prevalence reported by the authors refer to an increase? Was there an increase of 40.2% and 51.7% over the periods mentioned? | We adjusted the manuscript to present the results by sex and period of years. |
| Reviewer 2 | Results:  22. Table 1: Please include “n” for each category. | We included this information in the Table 1. |
| Reviewer 2 | Results:  23. Figure 1, title: Again, update the word “excess weight”. | This information was updated. |
| Reviewer 2 | Results:  24. S1 Figure was not cited in the Results section. Also, include the definition of “ES” in the S1, S2 and S3 Figures. | The figures presented in the Supporting information have been changed as appropriate. |
| Reviewer 2 | Results:  25. Page 6, line 144: Use only one type of denomination (sex: male/female or gender: men/women) throughout the manuscript, including tables and figures. | We adjusted the text.. |
| Reviewer 2 | Results:  26. Page 8, lines 143-46: “In almost all regions and in national surveys, except in North region, there was a predominance of male overweight compared to female”. This information is from Table 1, not S2 Fig. Add Table 1 at the end of the paragraph. | We adjusted the text to present the results by sex and period of years. |
| Reviewer 2 | Discussion: 27. Page 9, line 172: “We also observed a trend to publish results with high prevalence”. I wouldn’t say that! Is that a phenomenon that occurs only in Brazil? However, the authors need to better support this statement with more studies conducted in Brazil and in other countries. | The text was modified for better understanding. |
| Reviewer 2 | Discussion:  28. Page 9, line 185: “The results…”. Which results? | We included this information in the text. |
| Reviewer 2 | Discussion:  29. Page 10, line 191: “The prevalence of excess weight has been increasing.” In Brazil? Worldwide? Please mention! | We included this information in the text. |
| Reviewer 2 | Discussion:  30. Page 10, line 192: “…trend of increasing prevalence, …”. Which prevalence? Excess weight? Overweight? Obesity? | We included this information in the text. |
| Reviewer 2 | Discussion:  31. Page 10, line 197: “…in 2016; high values …” high or higher values? | We included this information in the text. |
| Reviewer 2 | Discussion:  32. Page 10, lines 197-98: “…In a study that evaluated different countries in Europe, the prevalence of overall excess weight was 53.1% and obesity, 15.9%, close to the Brazilian reality”. Were these studies carried out over the same period? | We included this information in the text. |
| Reviewer 3 | General issues  You seem to have lumped all data from 1974 to 2016 together into one meta-analysis, which yielded average prevalence data for this time period. I’m not sure how meaningfull it is to combine data from such a long time period, in particular given the enormous changes Brazil has undergone during this time period. For decicion-makers in policy and practice and researchers it would be more useful to know how high the prevalence was in 1974, and how high it was in 2016, rather than having an average figure for the whole time period. Please explain and consider doing separate meta-analyses for different time periods or points of time. | We performed new analyses considering sex and period of years. |
| Reviewer 3 | Abstract  Line 34-35 (“The overall prevalence of excess weight in Brazilian adult population was 46.7% (95% CI: 43.6; 49.7%).”): Please specify to which time point or time period this refers. | We included this information in the text. |
| Reviewer 3 | Introduction  Line 51-52 (“The consequences of being overweight… decrease life expectancy (7) due to an increased risk of premature death (1,8).“): Isn’t a decrease in life expectancy just another way of saying that the risk of premature death is increased? I find it somewhat strange to say that one is the consequence of the other. | We agree with this and have revised the information. “…due to increased risk of premature death” was excluded. |
| Reviewer 3 | Introduction  Line 53-54 (“Brazil is one of the few developing countries that conducts frequent national cross-sectional, population-based surveys..“): Wouldn’t it be more appropriate to describe Brazil as a middle-income country? | We agree with this and have incorporated your suggestion throughout the Introduction. |
| Reviewer 3 | Introduction  line 55-57 („Comparative results of some national surveys have shown an increase in the prevalence of excess weight in adults, an important event in the process of nutritional and socioeconomic transition“): I would frame the increase in the prevalence of excess weight as a development or process rather than an event. | This sentence was modified according to suggestion. |
| Reviewer 3 | Introduction  Line 57-59 (“This is not exclusive to the adult population, since there is a higher prevalence in children and adolescents, suggesting that the problem becomes even more serious over the years.“): Please provide a reference for the statement that the prevalence of obesity is hight among children and adolescents than among adults in Brazil (in most countries I am aware of it’s the other way round). | This sentence was modified, and references were included. The idea was shown that in Brazil, the prevalence of excess weight and obesity are increasing both in adults and in children. |
| Reviewer 3 | Introduction  Line 60-62 („Despite the numerous national surveys carried out, studies on prevalence of excess weight, overweight and obesity with Brazilian adults still so far fragmented and to the best of our knowledge, there are no previous systematic review that synthesized available data.): The NCDRisC Risk Factor Collaboration has published multiple pooled analyses of body weight trajectories, which include Brazil and which are based on reviews of existing surveys, see https://ncdrisc.org/publications.html. | Previous analysis only included national representative studies. Searching in the main databases doesn’t show studies that included local, regional, and national surveys. |
| Reviewer 3 | Methods  Line 67-70 (Protocol and registration): Please state if this was an a priori protocol, i.e. a protocol developed before the study began, and if there were any deviations from the protocol (i.e. anything you ended up doing differently than envisioned in the protocol – which is fine as long as it is stated transparently and reasonably justified). | This protocol was developed before the study began, but there were not important deviations from the protocol. Some changes were made regarding the search period. |
| Reviewer 3 | Methods  Line 71-73: I would propose to insert a separate sub-heading for the section on search methods (line 71-73). | We structured the text according to PRISMA checklist (2020). |
| Reviewer 3 | Methods  Eligibility criteria, line 76-77 („cross-sectional, population-based studies and random sampling, carried out in Brazil“): Please specify if studies had to be conducted on a national basis, i.e. if it was an eligibility criteria that they were representative for the whole of Brazil, or if you also included studies from specific regions within Brazil. | Previous analysis only included national representative studies.  We considered cross-sectional, population-based studies with random sampling carried out in Brazil (city, regional or national). |
| Reviewer 3 | Methods  Eligibility criteria, line 76-77 („cross-sectional, population-based studies and random sampling, carried out in Brazil“): Please specify if you also included cohort studies. | We considered longitudinal population-based studies that described the baseline of the study and not the segment. |
| Reviewer 3 | Methods  Line 95-97 (“The methodological quality of included studies was assessed using standardized tool The Joanna Briggs Institute Critical Appraisal Checklist for Studies Reporting Prevalence Data)“). As not all readers may be familiar with this tool, please consider providing a concise summary of it (2-3 sentences, and a table with the criteria used by the checklist). | We included this information in the text. |
| Reviewer 3 | Results  Line 116-117 („No new studies were identified by searching references of the included articles.“): Searching the reference lists of included studies is indeed recommended, and should also be mentioned in the methods section under the sub-heading „Search methods“. | This information was included in the Method section. |
| Reviewer 3 | Results  Line 125 („The overall prevalence of excess weight was 46.7%“). Please specify to which year this statement refers. This also applies to all similar statements in the subsequent sections of the manuscript. | This information was included in the text. |
| Reviewer 3 | Results  Line 131-13 („Trends on prevalence of excess weight has been increasing over the years (Fig 2).“): Figure 2 seems to show that the level of prevalence of obesity is increasing, which is not the same like saying that the trend on the prevalence of obesity is increasing (the latter seems to imply that the rate of chenge ist increasing. | We adjusted the manuscript and figure 2 to present the results by sex and years. |
| Reviewer 3 | Results  Line 132-134 („Between 1974 and 2004, the prevalence of excess weight was 40.2% (95% CI: 36.8; 43.7%) and from 2005 onwards, it has been 51.7% (95% CI: 48.8; 54.6%).“): To me, it seems a bit strange to lump such a long time period (1974-2004) together, and to say that from 2005 onwards, the prevalence has been exactly 51.7%. In reality, the prevalence seems to have been constantly rising. Besides, why have you chosen this specific cut-off (i.e. the year 2004/2005)? | We adjusted the manuscript to present the results by sex and period of years. |
| Reviewer 3 | Results  Table 1, as well as several instances throughout the manuscript: It seems as if you used the term „excess weight“ as summary category for both simple overweight (i.e. overweight without obesity) and obesity. Besides, you seem to use the category „overweight“ for simple overweight only (i.e. a BMI from 25 to 30), not including those with obesity. This should be made explicit somewhere. | This information was included in Method section. |
| Reviewer 3 | Results  line 141 („The overall prevalence of overweight in Brazil was 34.3%“): Please specify to which time period this refers. The same applies to similar statements in the following sections. Whenever a prevalence rate is given, the time frame should be stated explicitly. | This information was included in the text. |
| Reviewer 3 | Results  line 154-156 (“The excess weight prevalence funnel plot showed a high asymmetry (S4 Fig.). and a potential publication bias, which was confirmed by the presence of the effect of small studies (p = 0.001).“): Please state in which direction the data is likely to be biased (i.e. did small studies find, on average, higher prevalence rates than bigger studies, or was it the other way round?). | This information was included in the text. |
| Reviewer 3 | Discussion  Line 159 (“The prevalence of excess weight has been increasing since the mid-1970s.“): While it may be obvious, it may still be worthwhile stating that this sentence refers to Brazil. | This information was included in the text. |
| Reviewer 3 | Discussion  Line 185-188 („The results may also be influenced by survival bias. (…)“). Survival bias implies that the lifetime prevalence of obesity may be higher than the estimates for the point prevalence of obesity which you derived. However, in my understanding it does not impact the validity of the the latter – it just implies that they are lower than the lifetime prevalence. Please consider making this clearer in the discussion. | This information was included in the text. |
